# Quantitative evaluation of ocular vascularity and correlation analysis in patients with diabetic retinopathy by SMI and OCTA

**DOI:** 10.1186/s12886-024-03338-4

**Published:** 2024-02-19

**Authors:** Jin Wei, Chong Chen, Yinchen Shen, Fang Li, Shu Yiyang, Haiyun Liu

**Affiliations:** 1 Department of Ophthalmology, Shanghai General Hospital, Shanghai Jiao Tong University School of Medicine, Shanghai, 20080 China; 2 National Clinical Research Center for Eye Diseases, Shanghai, 20080 China; 3 Shanghai Key Laboratory of Ocular Fundus Diseases, Shanghai, 20080 China; 4 Shanghai Engineering Center for Visual Science and Photomedicine, Shanghai, 20080 China; 5 Shanghai engineering center for precise diagnosis and treatment of eye diseases, Shanghai, 20080 China; 6 Department of Ultrasound, Shanghai General Hospital, Shanghai Jiao Tong University School of Medicine, Shanghai, 20080 China; 7 Department of Ophthalmology, Tongji Hospital, School of Medicine, Tongji University, Shanghai, 200065 China

**Keywords:** SMI, Ultrawide-field OCTA, Diabetic retinopathy, Hemodynamics

## Abstract

**Aims:**

To find potential relation between retrobulbar vessels and fundus microvessels and to detect sensitive and effective clinical indicators in predicting the progress of diabetic retinopathy (DR), ocular hemodynamics were measured using superb microvascular imaging (SMI) and ultrawide-field optical coherence tomography angiography (UWF-OCTA).

**Methods:**

Observational, cross-sectional study evaluating ocular hemodynamics in patients with DR by SMI (Aplio i900, Canon Medical) and UWF-OCTA (BM-400 K BMizar, Tupai Medical Technology). The peak systolic velocity (PSV), end-diastolic velocity (EDV), and resistive index (RI) of the central retinal artery (CRA), posterior ciliary artery (PCA), and ophthalmic artery (OA) were measured by SMI. UWF-OCTA evaluated the fundus vascular parameters. A correlation analysis was used to determine the correlation between SMI and UWF-OCTA parameters.

**Results:**

One hundred thirty-nine eyes of 139 diabetic patients were included: 29 without DR (NDR), 36 with mild to moderate nonproliferative DR (M-NPDR), 37 with severe NPDR (S-NPDR), and 37 with proliferative DR (PDR). PSV and EDV of retrobulbar vessels decreased from NDR to S-NPDR while increasing PDR. RI of OA showed a decreasing trend in the progression of DR, but other vessels didn’t show the same trend. ROC curve analysis showed that CRA_PSV_, CRA_EDV_, PCA_EDV_, OA_PSV,_ and OA_EDV_ had diagnostic value distinguishing M-NPDR and S-NPDR. The correlation analysis observed a significant association between the SMI parameters of CRA and PCA and UWF-OCTA parameters. CRA hemodynamics were more associated with fundus vascular parameters, especially the retina, in the NDR group than in the M-NPDR group. In contrast, PCA consistently correlated with fundus vascular parameters, especially in the choroid, from the NDR to the M-NPDR group. However, OA showed a poor correlation with OCTA parameters.

**Conclusion:**

The velocity of retrobulbar vessels, mainly the CRA, may serve as a valuable predictor for assessing the progress of DR. The use of SMI in diabetic patients may help identify patients at risk of developing retinopathy.

**Supplementary Information:**

The online version contains supplementary material available at 10.1186/s12886-024-03338-4.

## Introduction

Diabetes mellitus (DM) is a rapidly increasing disease worldwide and causes macrovascular complications (cardiovascular) and microvascular complications (diabetic nephropathy, retinopathy, and neuropathy) [1]. Diabetic retinopathy (DR), a high prevalence of retinal microvascular complication, is globally a leading cause of vision loss in the working-age population in developed and developing nations [2]. Substantial evidence suggests that vascular self-regulation is impaired in the early stages of DR before the pathological changes, including retinal perfusion pressure and increased blood flow volume. The increased blood flow volume leads to an increase in shear force, which leads to vascular dilation, matrix increase, blood vessel wall damage, and blood vessel block. The inadequate oxygen supply to retinal tissue presumably causes tissue hypoxia, thus provoking angiogenesis. DR leads to poor circulation, a loss of visual function, and progress from non-proliferative diabetic retinopathy (NPDR) to proliferative diabetic retinopathy (PDR). Although vascular hemodynamics have been quietly changing, early retinal changes may not be detected on examination [3].

Previous studies have indicated that significant hemodynamic changes were associated with retinopathy progress [4]. They also identified that patients with a duration of diabetes of five years or less have shown a constriction of the major arteries and arterioles of retrobulbar circulation, and retinal blood flow is decreased [5]. During the prolongation of diabetes and/or retinopathy progression, arterial vasodilation occurs, and retinal blood flow increases in proportion to the severity of retinopathy [6]. Color Doppler flow imaging (CDFI) has been the most commonly used method, which can achieve noninvasive and direct measurement of retrobulbar vessels. The ophthalmic artery (OA), which is the first branch of the internal carotid artery (ICA) and changes with the hemodynamics of the CA [7], as well as the central retinal artery (CRA), the posterior ciliary arteries (PCA), supply blood and nutrition to the eyeball [8, 9]. Conflicting results about retrobulbar hemodynamics exist among different stages of DR; some studies found that the blood velocity and resistive index are statistically different in the control group than in the PDR group [4, 10, 11]. In contrast, others showed no significant alteration [12]. Owing to the poor repeatability and accuracy of CDFI for low-speed microflow detection, this biomarker has not been widely validated clinically, and no unified conclusion has been reached.

Superb microvascular imaging (SMI) is a novel Doppler ultrasound technology. Based on traditional Doppler ultrasound, its algorithm is updated to remove tissue motion and clutter artifacts and improve the sensitivity and accuracy of low-speed microvascular detection. At the same time, without using contrast agents, it maintains a high resolution of blood vessels, which can be used for early diagnosis of angiogenesis-related diseases [13, 14]. Ultrawide-field optical coherence tomography angiography (UWF-OCTA) allows visualization of peripheral and multilayer retinal and choroidal vasculature. OCTA is an essential auxiliary tool but cannot replace fundus fluorescein angiography (FFA) [15]. This approach is vulnerable to imaging artifacts and segmentation errors caused by corneal leukoma, cataracts, vitreous floaters, long axial length, silicone, and small pupils that cannot be dilated [16]. Besides, OCTA is not an actual flow; very low and high flows will be missed. Different devices have different strategies. It is essential to maintain a consistent approach in the long-term follow-up of DR patients [17]. These factors can lead to inaccuracies and limit its utility.

This article aimed to quantitatively analyze ocular vascularity by SMI and UWF-OCTA to determine the association between ocular fundus microvasculature and retrobulbar hemodynamics.

## Materials and methods

This study was approved by the institutional ethics committee of the Shanghai General Hospital affiliated with Shanghai Jiao Tong University and carried out by the tenets of the Declaration of Helsinki. All participants signed an informed consent form. Before formal enrolment, all patients underwent a comprehensive ocular examination, including slit lamp microscopy, visual acuity test, intraocular pressure examination, subjective refraction, axial length, wide-angle fundus photograph, fundus fluorescein angiography (FFA), and OCT. The diagnosis and severity of DR were determined according to the system used in the Early Treatment Diabetic Retinopathy Study [18]. The patients were then divided into four groups: patients with DM without DR, with mild to moderate NPDR, those with severe NPDR, and with PDR. The inclusion criteria were as follows: (1) type 2 diabetic patients; (2) diopter: -6.00 D ~ 6.00 D, axial length: 22 mm ~ 26 mm; and (3) no obvious refractive media opacification. The exclusion criteria were as follows: (1) previous history of fundus laser, intravitreal injection, traumatic eye diseases, or ocular surgery; (2) intraocular pressure > 21 mmHg or glaucoma; (3) any diseases other than diabetes or high blood pressure (systolic blood pressure < 160 mmHg, diastolic blood pressure < 100 mmHg) that can affect blood flow; (4) gross hypertensive retinopathy was ruled out clinically, concurrent or previous uveitis, other vascular and inflammatory retinal diseases; and poor fundus imaging quality due to opacification of refractive media(such as fibrotic stages of DR were excluded), inability to dilate pupils, inaccurate retinal segmentation (such as diabetic macular edema was excluded), artifacts or poor fixation stability (automatic signal intensity score < 6 out of 10) [16].

**Fig. 1:**
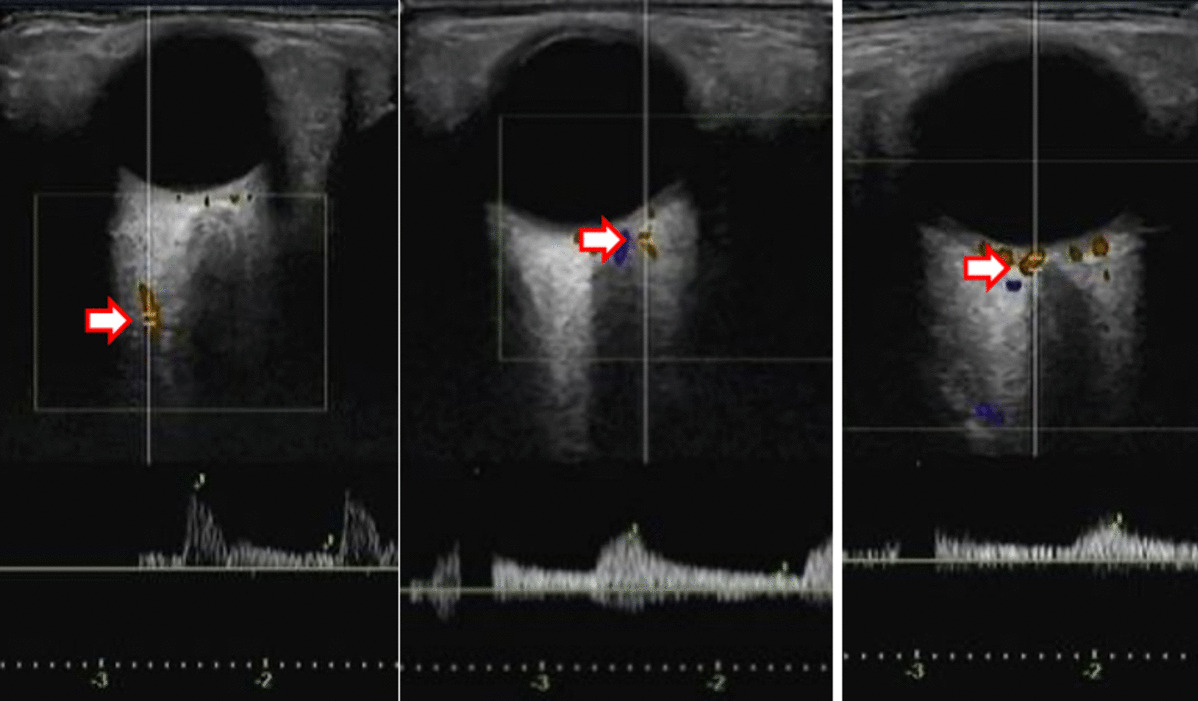
Retrobulbar blood vessels in SMI examination: **a** ophthalmic artery; **b** posterior ciliary artery; **c** central retinal artery

The retrobulbar blood vessels were detected using the Aplio i900 (Canon Medical Systems Corporation, Otawara, Touching, Japan) with a high-frequency linear probe [19]. All SMI examinations were performed by the same experienced sonographer (Anglia) to ensure repeatability [20]. Each patient was supine, and the probe was applied to the closed eyelid using sterile coupling gel. The probe was placed gently on the eyelids without any compression. The examined retrobulbar blood vessels were all located by reference to the optic nerve (Fig. 1). The central retinal artery (CRA) was found 10–15 mm away from the optical disc inside the optic nerve. The posterior ciliary artery (PCA) was observed near the lateral or medial optic nerve. The ophthalmic artery (OA) was usually found lateral to the optic nerve. The following hemodynamic parameters were measured in the blood mentioned above vessels: peak systolic blood velocity (PSV) (cm/s), end-diastolic blood velocity (EDV) (cm/s), and resistivity index (RI). The RI was then calculated using the expression [(PSV—EDV)/PSV] with a computer for each measured vessel.

OCTA was performed using the newly developed SS-OCT equipment (BM-400 K BMizar; TowardPi Medical Technology Co., Ltd., Beijing, China) with a laser light wavelength of 1060 nm, an acquisition speed of 400,000 A-scans/second, a bandwidth of 100 nm, and axial and transversal resolutions of 3.8 µM and 10 µM in tissues, respectively. The 24 mm × 20 mm area was centered on the fovea with motion correction. The split-spectrum amplitude-decorrelation angiography algorithm was used to automatically segment the superficial vascular complex (SVC) that ranges from the inner limiting membrane (ILM) to 9 µM above the inner plexiform layer (IPL), the deep vascular complex (DVC) that spans from 9 µM above the IPL to 9 µM beneath the outer plexiform layer (OPL), the choriocapillaris layer (ChC) that is from Bruch’s membrane to 29 µM beneath the membrane, and the choroidal vessel layer (ChV) that starts from 29 µM below Bruch’s membrane to the sclera. The quantitative indexes included the superficial vessel density (SVD), deep vessel density (DVD), choriocapillary layer vessel density (ChCVD), choroidal vessel layer vessel density (ChVVD), choroidal vessel volume index (CVI) and choroidal vessel volume (CVV). The vessel density is defined as the percentage of the vascularized area in an 18 × 18 mm area at a specific vascular layer by the binarized images. All the primary data were acquired and exported by the built-in software of the SS-OCT and SS-OCTA platforms.

Statistical analysis was performed using SPSS software (V.26.0). All numerical data are described as the mean ± SD. All enumeration data were investigated at the 95% confidence interval. Analysis of variance (ANOVA) was used for differences in the numerical data, with Bonferroni correction for multiple comparisons. The ROC curve evaluated the diagnostic sensitivity of different indicators between M-NPDR and S-NPDR. The Spearman correlation coefficient analyzed the correlation between the ocular fundus parameters and retrobulbar hemodynamics. A *p*-value of < 0.05 indicated a statistically significant difference.

## Results

A total of 139 eyes from 139 diabetic patients were included in this study. They were divided into four groups according to the EDTRS: diabetic patients without DR (*n* = 29 eyes, 20%), patients with M-NPDR (*n* = 36 eyes, 26%), patients with S-NPDR (*n* = 37 eyes, 27%) and patients with PDR (*n* = 37 eyes, 27%). The relevant characteristics of all the study participants are listed in Table 1. There were no significant differences between the groups regarding sex, eye type, or hypertension (*P* < 0.05). They can be pairwise comparisons. In terms of age and duration, there were significant differences among groups with the progression of DR (*P* = 0.004, *P* < 0.001) (Table 1).

**Table 1:** Characteristics of patients with different degrees of DR

	**Total**	**NDR**	**M-NPDR**	**S-NPDR**	**PDR**	***P* value**
Patients(n)	139	29	36	37	37	—
Gender (n [%])						
Male	82(59)	16(66)	20(56)	25(68)	18(49)	0.289
Female	57(41)	10(34)	16(44)	12(32)	19(51)	
Eye (n [%])						
Left	67(48)	14(48)	15(42)	20(54)	19(51)	0.884
Right	72(52)	15(52)	21(58)	17(46)	18(49)	
Hypertension (n [%])						
Yes	80(58)	8(28)	26(72)	22(59)	24(65)	0.611
No	59(42)	21(72)	10(28)	15(41)	13(35)	
Age(years)	57.32 ± 1.04	62.69 ± 1.82	59 ± 2.26	56.59 ± 1.82	52.19 ± 1.99	0.004
Durations(years)	10.67 ± 0.65	5.96 ± 0.76	13.53 ± 1.59	11.89 ± 1.01	10.35 ± 1.25	0.001
IOP (mmHg)	16.15 ± 2.22	15.87 ± 1.79	16.82 ± 2.19	16.37 ± 2.23	15.51 ± 2.40	0.068

**Fig. 2:**
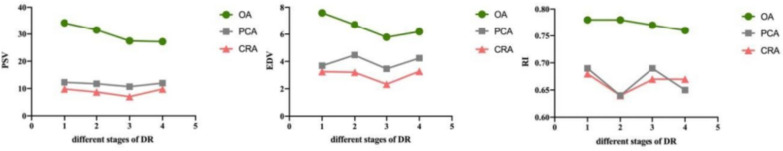
(1–3) Retrobulbar hemodynamics of different stages of DR (1: NDR, 2: M-NPDR, 3: S-NPDR, 4: PDR)

PSV, EDV, and RI of the OA, PCA, and CRA were measured and analyzed in retrobulbar blood vessels of DR patients in different stages. The results showed that with increasing DR severity, the PSV of OA, PCA, and CRA decreased from NDR to S-NPDR, remained the lowest in S-NPDR, and then increased in PDR. The EDVs of OA and CRA also increased first and then decreased, remaining the lowest in the S-NPDR group. The RI of OA decreased gradually as DR progressed, while no such change was observed in the other two vessels (Fig. 2). OA_PSV_, PCA_EDV_, PCA_RI_, CRA_PSV,_ and CRA_EDV_ were significantly different among the different groups (*P* = 0.008, *P* = 0.017, *P* = 0.007, *P* < 0.001, *P* < 0.001). CRA_PSV_ and CRA_EDV_ were significantly higher in the NDR group than in the S-NPDR group, and the difference was statistically significant (*P* < 0.001, *P* = 0.002); PCA_PSV_, PCA_EDV_, CRA_PSV_ and CRA_EDV_ were significantly lower in the NDR group than in the M-NPDR group (*P* = 0.026, *P* = 0.026, *P* = 0.012, *P* = 0.002). In contrast, the PCA_RI_ in the S-NPDR group was significantly higher than in the M-NPDR group (*P* = 0.026). In addition, CRA_PSV_ and CRA_EDV_ in the PDR group were significantly higher than those in the S-NPDR group (both *P* < 0.001) (Table 2). The association between IOP/MAP and retrobulbar hemodynamics was analyzed (Supplementary Table S3, Figs. S1 and S2).

**Table 2:** Ocular hemodynamic parameters of eyes with DR in different stages

	**NDR**	**M-NPDR**	**S-NPDR**	**PDR**	***P* value**	***P* value**
Opthalmic artery (OA)						
PSV (cm/s)	34.21 ± 2.29	31.55 ± 1.69	27.49 ± 11.81	27.23 ± 1.62	0.008*	0.016†(NDR-MNPDR)
EDV (cm/s)	7.59 ± 0.81	6.71 ± 0.37	5.78 ± 0.42	6.19 ± 0.31	0.062	—
RI	0.78 ± 0.01	0.78 ± 0.01	0.77 ± 0.10	0.76 ± 0.10	0.37	—
Posterior ciliary artery (PCA)						
PSV (cm/s)	12.27 ± 0.58	11.76 ± 0.75	10.69 ± 0.53	11.99 ± 0.63	0.303	0.026†(MNPDR-SNPDR)
EDV (cm/s)	3.69 ± 0.16	4.47 ± 0.28	3.46 ± 0.23	4.24 ± 0.29	0.017*	0.026†(MNPDR-SNPDR)
RI	0.69 ± 0.11	0.64 ± 0.01	0.69 ± 0.01	0.65 ± 0.01	0.007*	0.026†(MNPDR-SNPDR)
Central retinal artery (CRA)						
PSV (cm/s)	9.85 ± 0.36	8. 73 ± 0.32	7.01 ± 0.36	9.81 ± 0.50	< 0.001*	< 0.001†(NDR-SNPDR) 0.012†(MNPDR-SNPDR) < 0.001†(SNPDR-PDR)
EDV (cm/s)	3.26 ± 0.17	3.21 ± 0.17	2.34 ± 0.13	3.28 ± 0.20	< 0.001*	0.002†(NDR-SNPDR) 0.002†(MNPDR-SNPDR) < 0.001†(SNPDR-PDR)
RI	0.68 ± 0.01	0.64 ± 0.01	0.67 ± 0.01	0.67 ± 0.01	0.154	—

*PSV* peak systolic blood velocity(cm/s), *EDV* end-diastolic blood velocity (cm/s), *RI* resistivity index

^*^indicates a significant difference among different groups

^†^indicates a statistically significant difference between the groups, and *P* < 0.05 was considered statistically significant

**Fig. 3:**
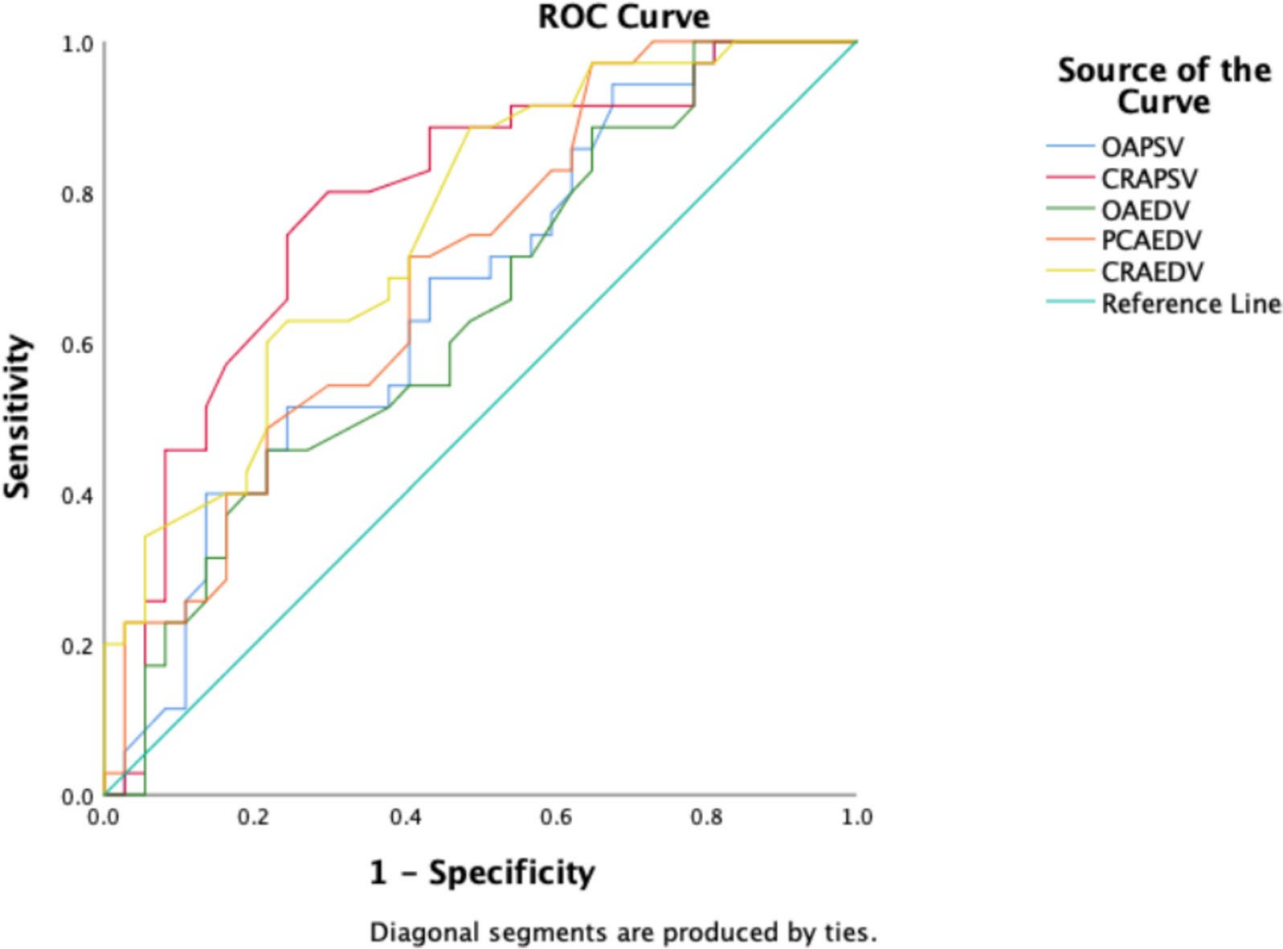
ROC curves of ocular hemodynamics between M-NPDR and S-NPDR

Table 3 summarizes the diagnostic sensitivity of the hemodynamic indexes of CRA, PCA, and OA between M-NPDR and S-NPDR by ROC curve. Among all the hemodynamic parameters, the area under the curve (AUC) of CRA_PSV_ was the highest (0.781), but the sensitivity and specificity were lower (80% and 30%, respectively). PCA_PSV_ had relatively high sensitivity (94%) and the highest specificity (68%) among these indexes. The sensitivity and specificity of PCA_EDV_ were 97% and 65%, respectively. CRA_EDV_ and OA_EDV_ had the same sensitivity (89%) in this diagnostic prediction model, but the specificity of CRA_EDV_ (49%) was slightly lower than OA_EDV_ specificity (65%) (Table 3 and Fig. 3). In addition, retrobulbar hemodynamic parameters with statistical differences in ROC analysis for VTDR vs NDR were analyzed (Supplementary Table S4. and Fig. S3). The AUC of these indexes was all below 0.7, which means the diagnostic accuracy is a bit lower than that for M-NPDR vs S-NPDR.

**Table 3:** Receiver curves of retrobulbar hemodynamic parameters between M-NPDR and S-NPDR

Retrobulbar hemodynamics	Area Under Curve	Sensitivity, %	Specificity, %	Cut-off, cm/s
CRA_PSV_	0.781	80	30	10.10
CRA_EDV_	0.754	89	49	1.9
PCA_EDV_	0.696	97	65	3.7
OA_PSV_	0.661	94	68	27.1
OA_EDV_	0.638	89	65	5.7

Furthermore, retinal and choroid blood flow data from 74 eyes in 74 patients were analyzed and divided into three groups, including 20 eyes in the NDR group, 32 in the M-NPDR group, and 22 in the S-NPDR/PDR group. Retinal and choroid blood flow parameters in different groups were summarized and analyzed. Some statistically significant data are shown (Supplementary Table S1 and S2).

The correlation analysis results of ocular vascularity in SMI and UWF-OCTA parameters are presented below. The retrobulbar parameters in SMI were significantly correlated with retinal and choroidal parameters in different degrees of DR. The blood velocity CRA was correlated with fundus vascular density in the NDR group. CRA_PSV_ was positively correlated with SVD (15–18 mm), ChCVD, and ChVVD in some quadrants; CRA_EDV_ was positively correlated with SVD (3–6 mm/15-18 mm), DVD (0–6/6–9/15-18 mm), and ChVVD in some quadrants (all *P* < 0.05). Meanwhile, CRA_RI_ was negatively associated with SVD (3–6 mm), specific area of DVD (0–6/6–9/15-18 mm) (all *P* < 0.05). No significant correlation was found between CRA_RI_ and choroidal parameters (Table 4). In the M-NPDR group, no correlation was found between the hemodynamic parameters of CRA and retinal parameters. However, CRA_PSV_ was positively correlated with temporal ChCVD, and CRA_EDV_ was correlated with nasal ChVVD with statistical significance (Table 5). In the S-NPDR/PDR group, there was no correlation between CRA_EDV_ and CRA_RI_ and fundus parameters. The association was only found between CRA_PSV_ and temporal SVD (6–9 mm) (*P* < 0.05) (Table 6).

**Table 4:** Correlation analysis of retinal and choroid parameters and retrobulbar hemodynamics in the NDR group

NDR	CRA_PSV_	CRA_EDV_	CRA_RI_	PCA_PSV_	PCA_EDV_	PCA_RI_	OA_PSV_
**Retinal vascular density/SVD (%)**							
1–3 mm	-	-	-	-	-	-0.672 ~ -0.668^a^	-
3–6 mm		0.752^a^	-0.755^a^	0.794^b^	0.842^b^	-	-
12–15 mm	-	-	-	-	-	-	-0.643^a^
15–18 mm	0.673 ~ 0.788^a^	0.782^b^	-	-	-	-	-
**Retinal vascular density/DVD (%)**							
0–1 mm	-	0.758^a^	-0.687^a^	0.710^a^	0.758^a^	-	-
1-6 mm	-	0.665 ~ 0.705^a^	-0.899 ~ -0.635^b^	-	-	-	-
6–9 mm	-	0.681 ~ 0.693^a^	-0.855 ~ -0.788^b^	-	-	-	-
15–18 mm	-	0.758^a^	-0.681^a^		0.697^a^	-	-
**Vascular density of ChC (ChCVD)**							
**Some quadrants**	0.663^a^	-	-	-	-	-0.735 ~ -0.675^a^	-
**Vascular density of ChV (ChVVD)**							
**Some quadrants**	0.634 ~ 644^a^	0.669 ~ 0.695^a^	-	-	0.705^a^ ~ 0.835^b^	-	-0.681 ~ -0.671^a^
**CVV**	-	-	-	0.636^a^	0.636^a^	-	-

^a^indicates correlation coefficient r, with significant difference, *P* < 0.05

^b^indicates correlation coefficient r, with significant difference, *P* < 0.01

**Table 5:** Correlation analysis of retinal and choroid parameters and retrobulbar hemodynamics in the M-NPDR group

M-NPDR	CRA _PSV_	CRA _EDV_	PCA _PSV_	PCA _EDV_	PCA _RI_	OA _PSV_	OA _EDV_	OA _RI_
**Retinal vascular density/SVD (%)**								
1–3 mm	-	-	0.503^a^	0.499 ~ 0.709^b^	-	-	-	-
3–6 mm	-	-	-	-	-	-0.510^a^	-0.528^a^	-
6–9 mm	-	-	-	-	-	-0.575^a^	-	-
9–12 mm	-	-	0.511 ~ 0.688^b^	-	-	-	-	-
12–18 mm	-	-	0.504 ~ 0.792^b^	0.498 ~ 0.617^a^	-	-	-	-
**Retinal vascular density/DVD (%)**								
9–12 mm	-	-	-	-	-	-	-0.631 ~ -0.556^a^	-
12–15 mm	-	-	0.544^a^	-	-	-	-	-
15–18 mm	-	-	0.499 ~ 0.527^a^	0.560 ~ 0.721^b^	-	-	-	-
**Vascular density of ChC (ChCVD)**								
**Some quadrants**	0.543^a^	0.501^a^	-	-	-0.757 ~ -0.570^b^	-	-	-0.594^a^
**Vascular density of ChV (ChVVD)**								
**Some quadrants**	-	0.498^a^	-	-	-	-0.511^a^	-	-0.584 ~ -0.514^a^
**CVV**	-	-	-	0.579^a^	-	-	-	-
**CVI**	-	-	-	0.612 ~ 0.729^b^	-	-	-	-

^a^indicates correlation coefficient r, with significant difference, *P* < 0.05

^b^indicates correlation coefficient r, with significant difference, *P* < 0.01

**Table 6:** Correlation analysis of retinal and choroid parameters and retrobulbar hemodynamics in the S-NPDR/PDR group

S-NPDR/PDR	CRA_PSV_	PCA_PSV_	PCA_EDV_	PCA_RI_	OA_EDV_
**Retinal vascular density/SVD (%)**					
6–9 mm/temporal	0.635^a^	-	-	-	-
15–18 mm	-	-	0.654^a^ ~ 0.758^b^	-0.924^b^ ~ -0.724^a^	-
**Retinal vascular density/DVD (%)**					
0–1 mm	-	-	0.645^a^	-	-
1–3 mm	-	-	-	-0.712 ~ 0.654^a^ ~	-
9–12 mm	-	-	-	-0.641^a^ ~ -0.618^a^	-
**ChVVD** (Superior)	-	0.691^a^	-	-	-0.631^a^

^a^indicates correlation coefficient r, with a significant difference, *P* < 0.05

^b^indicates correlation coefficient r, with a significant difference, *P* < 0.01

For the PCA, in the NDR group, the velocity of PCA was positively correlated with SVD (15–18 mm), DVD (0–1 mm), and CVV (*P* < 0.05). PCA_RI_ was negatively correlated with SVD (1–3 mm) and ChCVD in some quadrants (*P* < 0.05) (Table 4). In the M-NPDR group, PCA_PSV_ was positively correlated with SVD (1–3 mm/9–18 mm) and DVD (12–18 mm) (all *P* < 0.05). PCA_EDV_ was significantly correlated with SVD (1–3 mm/12–18 mm) and DVD (15–18 mm) (all *P* < 0.05). In the correlation analysis with choroid parameters, PCA_EDV_ was positively associated with CVV and CVI (*P* < 0.05) but not found in PCA_PSV_. There was a negative correlation between PCA_RI_ and ChCVD (*P* < 0.05) (Table 5). In S-NPDR/PDR group, PCA_PSV_ was positively correlated with ChVVD; PCA_EDV_ was positively correlated with SVD (9–18 mm) and DVD (0–1 mm); PCA_RI_ was negatively correlated with SVD (9–18 mm), DVD (1–3 mm/6–12 mm) (*P* < 0.05) (Table 6).

For the OA, in the NDR group, OA_PSV_ was negatively correlated with some quadrants of SVD (12–15 mm) and ChVVD (*P* < 0.05) (Table 4). In the M-NPDR group, OA_PSV_ was negatively correlated with some quadrants of SVD (3–9 mm) and ChVVD (*P* < 0.05). OA_EDV_ was negatively correlated with SVD (3–6 mm) and DVD (9–12 mm), and OA_RI_ was negatively correlated with ChCVD (*P* < 0.05) (Table 5). In the S-NPDR/PDR group, only OA_EDV_ was negatively correlated with superior ChVVD (*P* < 0.05) (Table 6).

## Discussion

To the best of our knowledge, this is the very first study to evaluate retrobulbar hemodynamics by SMI and analyze its correlation with fundus microvessels parameters by UWF-OCTA in diabetic patients with DR. Our results showed that the retrobulbar hemodynamics and the strength of the associations varied at different stages of DR.

### Descriptive analysis

Substantial evidence indicated that when the diameter of the retinal artery in NDR patients has not changed [4], the retrobulbar vessels have already presented a state of low blood flow velocity and high resistance. This may further lead to retinal ischemia and hypoxia, blood vessel occlusion, and nonperfusion area formation, leading to the formation of S-NPDR. Previous studies have shown that the flow velocity of CRA in the diabetes group (including M-NPDR, S-NPDR, and PDR) was lower than that in the control group [21–24], which was also lower in the diabetes group (S-NPDR and PDR) compared with the control (NDR and M-NPDR) group [25]. Baydar et al. indicated that CRA_EDV_ in the NDR group was higher than in the M-NPDR group [23]. This study showed that CRA_PSV_ and CRA_EDV_ were the lowest in the S-NPDR group. Moreover, in the diagnosis model from M-NPDR to S-NPDR, the AUC of CRA_PSV_ and CRA_EDV_ (reaching 0.7) was significantly higher than other indexes. However, this study found no difference in CRA_RI_ between different diabetic groups, which may result from the limited sample size.

The current literature shows that the velocity of PCA in the diabetic group was lower than in the control group [21, 22]. These were all comparisons with healthy controls. This study found that PCAPSV and PCAEDV in S-NPDR were significantly lower than in M-NPDR, with significant differences. Gracner et al. also found that PCA_RI_ in the SNPDR/PDR group was higher than in the M-NPDR and control groups, similar to our results. We concluded that PCA_RI_ in the S-NPDR was significantly higher than in the M-NPDR. PCA supplies the outer layer of the retina and choroid, reflecting the microcirculation status of the retina and choroid to a certain extent. Our results showed that PCA has a similar trend as CRA, which is valuable for understanding DR's occurrence and development.

Mendivil et al. indicated that the velocity of OA in M-NPDR and PDR were lower than that in the control group using CDFI, respectively [26]. However, another study found that the OA_PSV_ in SNPDR/PDR was higher than in the control group [21]. We grouped DR in more detail and found that the OA_PSV_ in the M-NPDR group was significantly lower than in the NDR group. MacKinnon et al. found that OA_RI_ in both NDR and M-NPDR were more significant than those in the control group [24], and our results identified that RI gradually decreased in the development of DR to the lowest level in PDR.

### Correlational analysis

A recent study evaluated ocular vascularity in patients with myopic anisometropia using CDFI and OCTA and found a correlation between CRA_PSV_ and retinal SVD and DVD [27], consistent with our results. The retina's superficial and deep vascular complex is mainly distributed in the inner five retinal layers, the same as CRA. In the early stage of DR, the microcirculation was relatively normal. With the aggravation of fundus microvessels, many superficial and deep retinal blood vessels were blocked. Then, the blood flow of CRA could not reach the retina that should be achieved. However, in the M-NPDR group, the blood flow velocity was still correlated with choroidal parameters. In the first part of this study, it was found that the flow velocity of CRA also showed a downward trend with the progression of DR. For the M-NPDR group, there was no correlation between the fundus microcirculation and resistance index, which further confirmed our hypothesis that the hemodynamic indexes of CRA were significantly correlated with the retinal and choroidal parameters in early DR.

The blood flow velocity of PCA was mainly positively correlated with SVD (15–18 mm) and DVD (0–1 mm) in the NDR group. With the development of DR, the blood flow velocity was increasingly related to retinal VD, mainly in the temporal and inferior retina. Unlike CRA, the correlation between PCA_EDV_ and retinal VD could still be observed in the S-NPDR/PDR group. We measured only one branch of the posterior long ciliary artery, so the blood flow velocity was more correlated with the retina of the region it supplied. The hemodynamics of PCA were associated with choroidal parameters (including ChCVD, ChVVD, CVI, and CVV) at the early stage of DR. Still, the association persisted as the disease progressed and completely lost correlation in the S-NPDR/PDR group. Liu et al. found a correlation between choroidal parameters and PCA blood flow velocity in patients with myopia [27]. Compared with the correlation between blood flow velocity and retinal VD, the correlation between blood flow velocity and choroidal parameters (CVI and CVV) was more significant. The CVI is equivalent to the three-dimensional vessel density, representing the density of choroidal vessels. It means the ratio of choroid vessel volume to choroid volume within a designated three-dimensional region. The larger the value is, the denser the vessels. The CVV reflects the choroid vessel volume per unit area. The larger the value is, the more vessels there are [28]. According to our results, EDV is the most stable among the three indexes of PCA under SMI. Relatively speaking, PCA_EDV_ may be used to predict the progression of DR and may be used as a long-term clinical observation indicator for patients with DR.

Regarding the OA, only PSV was negatively correlated with nasal SVD and ChVVD in the NDR group. The association persisted until the S-NPDR/PDR. This may be because OA comes from the internal carotid artery and emits many branches, including CRA and PCA. Unlike the CRA, the OA needs to supply nutrients throughout the eyeball. Therefore, the correlation between the hemodynamics of OA and fundus parameters is not obvious. Furthermore, these correlations have no clear pattern, probably because of the small sample size. Previous studies have found that retinal VD, FAZ, and other fundus parameters did not change significantly after anti-VEGF treatment in patients with DME, while the retrobulbar vessel velocity decreased [29].

The above results indicated that the flow velocity of retrobulbar vessels has a specific diagnostic value from the M-SNPDR to S-NPDR. Although the specificity needs to be improved, the sensitivity was relatively high, which is valuable in the screening of DR. In addition, we identified that CRA was closely related to retinal VD, and PCA was closely related to choroidal parameters. Therefore, SMI can be a clinical biomarker to synergize in observing disease progression or curative effect with its high sensitivity when OCTA does not work.

### Limitations

The study has several limitations. First, due to the limitations of cross-sectional, long-term longitudinal observation of DR patients is needed to determine whether these indicators have accurate predictive value. Second, the sample size was relatively small, although the SMI parameters significantly differed. A larger sample size in different stages is required for further studies. Third, SMI is currently only used in ophthalmology for blood flow detection, such as retinoblastoma and optic nerve papilla [19], but has not been used in DR. SMI performs better in the clinical application of neovascularization compared with traditional ultrasound technology [30, 31], which makes its results more reliable. SMI should be evaluated more and considered for widespread application in ophthalmology.

## Conclusion

In summary, the present study provides explicit evidence that retrobulbar hemodynamics changes with the development of DR, and retrobulbar hemodynamic parameters changed most significantly from M-NPDR to S-NPDR, with CRA showing the highest performance. It is speculated that retrobulbar hemodynamics may be a clinical biomarker to predict advanced DR. Using SMI may help identify diabetic patients at risk of developing retinopathy and may be considered in the clinic.

### Supplementary Information

**Additional file 1: Supplementary Table S1. **Comparison of retinal blood flow parameters in UWF-OCTA in patients with different stages of DR.** Supplementary Table S2. **Choroidal blood flow parameters in UWF-OCTA in patients with different stages of DR.** Supplementary Table S3. **Correlation analysis of retrobulbar hemodynamics and IOP, MAP.** Supplementary Fig. S1. **Scatter plots between IOP and retrobulbar hemodynamics.** Supplementary Fig. S2. **Scatter plots between MAP and retrobulbar hemodynamics.** Supplementary Table S4. **Receiver curves of retrobulbar hemodynamic parameters between NDR and VTDR.** Supplementary Fig. S3. **ROC curves of ocular hemodynamics between NDR and VTDR.

## Data Availability

No datasets were generated or analysed during the current study.
