# Supplementary material for: Quantitative evaluation of ocular vascularity and correlation analysis in patients with diabetic retinopathy by SMI and OCTA
Source: BMC Ophthalmol. 2024 Feb 19;24:76. doi: 10.1186/s12886-024-03338-4 (PMC10875800; doi:10.1186/s12886-024-03338-4)
Supplement: Supplementary file 1 — Additional file 1: Supplementary Table S1. Comparison of retinal blood flow parameters in UWF-OCTA in patients with different stages of DR. Supplementary Table S2. Choroidal blood flow parameters in UWF-OCTA in patients with different stages of DR. Supplementary Table S3. Correlation analysis of retrobulbar hemodynamics and IOP, MAP. Supplementary Fig. S1. Scatter plots between IOP and retrobulbar hemodynamics. Supplementary Fig. S2. Scatter plots between MAP and retrobulbar hemodynamics. Supplementary Table S4. Receiver curves of retrobulbar hemodynamic parameters between NDR and VTDR. Supplementary Fig. S3. ROC curves of ocular hemodynamics between NDR and VTDR. [file 12886_2024_3338_MOESM1_ESM.docx]

**Supplementary Table S1 Comparison of retinal blood flow parameters in UWF-OCTA in patients with different stages of DR**

| **Parameters** | **NDR**  **n=20** | **M-NPDR**  **n=32** | **S-NPDR/**  **PDR**  **n=22** | **P value*** | **P value**† |
| --- | --- | --- | --- | --- | --- |
| **Superficial vascular density (SVD)/%** | | | | | |
| 12-15 mm// nasal | 32.9±2.25 | 30.31±1.99 | 23.3±2.64 | 0.027 | 0.03(NDR-SNPDR/PDR) |
| **Deep vascular density（DVD）/%** | | | | | |
| 1 mm/circle | 24.0±1.40 | 22.81±1.35 | 17.0±2.34 | 0.019 | 0.027(NDR-SNPDR/PDR) |
| 1-3 mm/nasal | 29.45±0.79 | 29.75±1.1 | 22.3±2.63 | 0.009 | 0.005(NDR-M-NPDR)  0.13 （NDR-SNPDR/PDR) |
| 1-3 mm/superior | 27.73±1.57 | 28.25±1.24 | 22.0±2.78 | 0.045 | — |
| 1-3 mm/temporal | 28.73±1.47 | 28.56±0.94 | 21.8±2.53 | 0.008 | 0.013(NDR-M-NPDR)  0.020(NDR-SNPDR/PDR) |
| 1-3 mm/inferior | 27.73±1.45 | 28.13±1.16 | 21.8±2.60 | 0.026 | 0.033(NDR-M-NPDR) |
| 3-6 mm/nasal | 27.36±1.34 | 27.81±1.04 | 21.5±2.12 | 0.009 | 0.011(NDR-M-NPDR) |
| 3-6 mm/temporal | 27.91±1.48 | 28.13±0.92 | 22.2±2.22 | 0.015 | 0.021(NDR-M-NPDR)  0.046(NDR-SNPDR/PDR) |
| 6-9 mm/inferior | 24.45±1.72 | 26.38±0.77 | 22.22±1.59 | 0.040 | — |
| 9-12 mm/inferior | 25.27±1.99 | 27.13±0.89 | 21.3±2.43 | 0.038 | — |
| 12-15 mm/inferior | 28.55±1.88 | 29.94±1.05 | 23.5±1.42 | 0.009 | 0.008(NDR-MNPDR) |

* indicates a significant difference among different groups;

† indicates a statistically significant difference between the groups, and P<0.05 was considered statistically significant.

**Supplementary Table S2 Choroidal blood flow parameters in UWF-OCTA in patients with different stages of DR**

| **Parameters** | **NDR**  **n=20** | **M-NPDR**  **n=32** | **S-NPDR/PDR**  **n=22** | **P P-value** |
| --- | --- | --- | --- | --- |
| **Vascular density of ChC（ChCVD）/%** | | | | |
| **Supratemporal** | 53.5±3.30 | 51.13±2.54 | 56.36±3.18 | 0.442 |
| **Superior** | 55.0±3.68 | 51.44±2.73 | 56.82±3.79 | 0.480 |
| **Supranasal** | 53.5±3.78 | 51.69±2.60 | 55.82±3.58 | 0.649 |
| **Temporal** | 49.60±3.72 | 49.13±2.98 | 54.36±3.55 | 0.499 |
| **Central** | 51.1±3.94 | 49.94±2.62 | 54.27±3.49 | 0.619 |
| **Nasal** | 50.0±4.38 | 50.31±3.35 | 54.55±3.57 | 0.654 |
| **Inferotemporal** | 51.4±4.97 | 72.63±11.99 | 58.73±6.19 | 0.298 |
| **Inferior** | 56.8±4.74 | 63.88±8.60 | 68.82±6.74 | 0.597 |
| **Inferonasal** | 57.0±6.42 | 67.50±8.84 | 67.18±6.73 | 0.619 |
| **Vascular density of ChV（ChVVD）/%** | | | | |
| **Supratemporal** | 60.5±5.34 | 75±10.25 | 61.09±5.02 | 0.372 |
| **Superior** | 67.9±6.07 | 69.38±8.90 | 72.27±5.64 | 0.933 |
| **Supranasal** | 64±8.06 | 68±8.44 | 58.45±4.92 | 0.677 |
| **Temporal** | 63.3±12.83 | 56.94±5.42 | 56.09±4.56 | 0.792 |
| **Central** | 70.1±14.52 | 58.0±6.38 | 59.73±5.77 | 0.606 |
| **Nasal** | 69.7±15.24 | 62.06±6.22 | 56.45±5.58 | 0.631 |
| **Inferotemporal** | 42.6±6.68 | 46.63±4.66 | 43.73±5.89 | 0.863 |
| **Inferior** | 41.1±5.73 | 46.19±4.52 | 45.09±5.39 | 0.777 |
| **Inferonasal** | 42±5.88 | 46.19±4.04 | 42.09±6.0 | 0.787 |
| **Choroidal Vessel volume（CVV）/μm** | | | | |
| **Supratemporal** | 52.1±10.11 | 62.75±9.25 | 54.45±9.94 | 0.635 |
| **Superior** | 47.6±6.63 | 53.19±8.42 | 51.09±8.76 | 0.895 |
| **Supranasal** | 41.9±6.48 | 58.13±9.12 | 47.18±10.20 | 0.430 |
| **Temporal** | 41.1±6.45 | 46.63±7.37 | 49.18±11.74 | 0.828 |
| **Central** | 39.5±6.03 | 53±11.40 | 49.18±9.11 | 0.646 |
| **Nasal** | 34.4±4.79 | 50.81±10.06 | 41.90±8.07 | 0.429 |
| **Inferotemporal** | 113.8±30.94 | 114.81±19.45 | 114.73±24.37 | 0.943 |
| **Inferior** | 116.2±31.08 | 111.81±19.84 | 122±24.70 | 0.955 |
| **Inferonasal** | 104.2±31.36 | 101.12±22.23 | 121.82±28.13 | 0.842 |
| **Choroidal Vessel Volume Index（CVI）/%** | | | | |
| **Supratemporal** | 97.1±28.70 | 95.5±23.50 | 106.82±27.92 | 0.949 |
| **Superior** | 99.8±28.72 | 93.38±23.68 | 108.91±26.47 | 0.911 |
| **Supranasal** | 92.2±27.46 | 99.44±25.42 | 111±30.15 | 0.904 |
| **Temporal** | 77.1±21.4 | 87.81±25.02 | 99.64±28.02 | 0.850 |
| **Central** | 79.3±21.45 | 88.75±24.42 | 102±28.69 | 0.844 |
| **Nasal** | 79.7±22.41 | 91.94±25.62 | 105.36±30.63 | 0.826 |
| **Inferotemporal** | 49.2±8.61 | 44.13±5.37 | 50.27±7.65 | 0.780 |
| **Inferior** | 47.3±8.63 | 45.06±5.90 | 48.09±7.13 | 0.946 |
| **Inferonasal** | 44.8±9.02 | 40.94±6.14 | 48.27±7.75 | 0.770 |

**Supplementary Table S3 Correlation analysis of retrobulbar hemodynamics and IOP, MAP**

| **M-NPDR** | **IOP** | **P_IOP_** | **MAP** | **P_MAP_** |
| --- | --- | --- | --- | --- |
| **CRA_PSV_** | 0.007 | - | -0.089 | - |
| **CRA_EDV_** | 0.042 | - | -0.178* | 0.037 |
| **CRA_RI_** | -0.169* | 0.048 | 0.173* | 0.044 |
| **PCA_PSV_** | 0.068 | - | 0.014 | - |
| **PCA_EDV_** | 0.022 | - | -0.11 | - |
| **PCA_RI_** | 0.036 | - | 0.093 | - |
| **OA_PSV_** | -0.042 | - | 0.045 | - |
| **OA_EDV_** | -0.049 | - | 0.081 | - |
| **OA_RI_** | 0.01 | - | -0.048 | - |


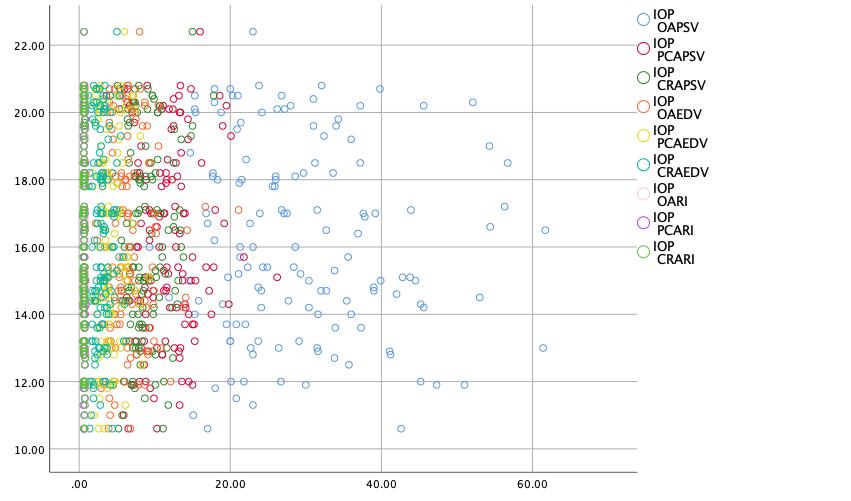


**Supplementary Fig.S1 Scatter plots between IOP and retrobulbar hemodynamics**


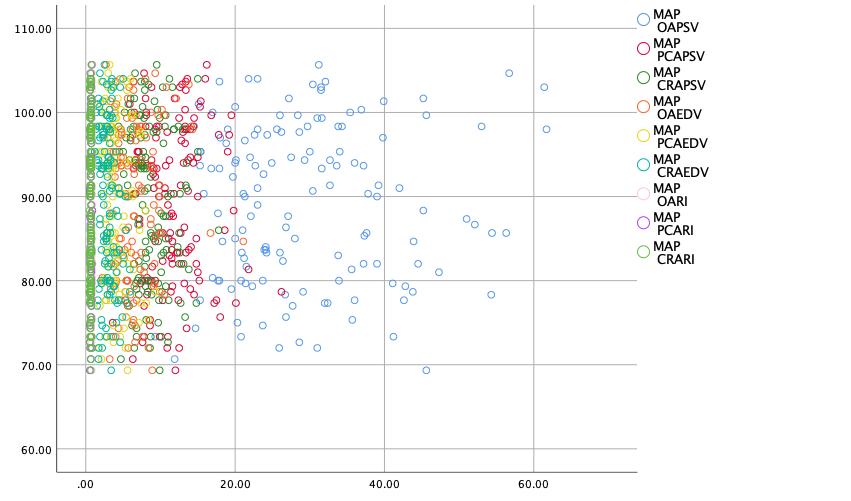


**Supplementary Fig.S2 Scatter plots between MAP and retrobulbar hemodynamics**

**Supplementary Table S4 Receiver curves of retrobulbar hemodynamic parameters between NDR and VTDR**

| **Retrobulbar hemodynamics** | **Area Under**  **Curve** | **Sensitivity, %** | **Specificity, %** | **Cut-off, cm/s** |
| --- | --- | --- | --- | --- |
| **CRA_PSV_** | 0.699 | 86.2 | 41.4 | 7.95 |
| **CRA_EDV_** | 0.643 | 89 | 49 | 2.15 |
| **OA_PSV_** | 0.683 | 94 | 68 | 33.25 |


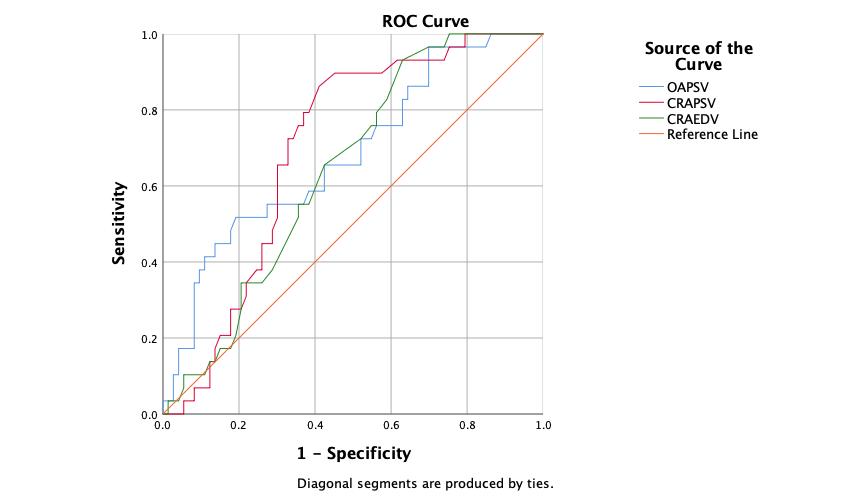


**Supplementary Fig.S3 ROC curves of ocular hemodynamics between NDR and VTDR**
